# Time- and Ventricular-Specific Expression Profiles of Genes Encoding Z-Disk Proteins in Pressure Overload Model of Left Ventricular Hypertrophy

**DOI:** 10.3389/fgene.2018.00684

**Published:** 2019-01-07

**Authors:** Anastasia Knyazeva, Alexander Krutikov, Alexey Golovkin, Alexander Mishanin, Georgii Pavlov, Natalia Smolina, Anastasia Hushkina, Thomas Sejersen, Gunnar Sjoberg, Mikhail Galagudza, Anna Kostareva

**Affiliations:** ^1^Almazov National Medical Research Centre, Saint Petersburg, Russia; ^2^Saint Petersburg State Academy of Veterinary Medicine, Saint Petersburg, Russia; ^3^Information Technologies and Programming Faculty, ITMO University, Saint Petersburg, Russia; ^4^Department of Women’s and Children’s Health, Center for Molecular Medicine, Karolinska Institute, Stockholm, Sweden

**Keywords:** Z-disk, cardiac hypertrophy, mechanotransduction, gene expression, pressure overload

## Abstract

Mechanotransduction is an essential mechanism of transforming external mechanical stimulus to biochemical response. In cardiomyocytes mechanotransduction plays an important role in contraction, stretch sensing and homeostasis regulation. One of the major mechanosensitive area in cardiomyocytes, the Z-disk, consists of numbers of structural and signaling proteins, that may undergo conformational or gene expression changes under pathological stress conditions. In present study we examined a rat model of pressure overload cardiac hypertrophy validated by echocardiographic and histopathological examinations. We revealed, that during hypertrophy progression expression of several genes encoding Z-disk proteins (*Actn2*, *Ldb3*, *Cmya5*, *Nebl*) is different at early and late points of cardiac remodeling. Moreover, expression patterns of several genes are opposite in myocardium of overloaded left ventricle and hemodynamically unaffected right ventricle, and expression profiles in interventricular septum are more similar to right ventricle. Additionally, we revealed inconsistencies between mRNA and protein level changes of Actn2, one of the major structural Z-disk element. All these findings point out, that investigated Z-disk proteins participate in pathological stress adaptation through undergoing the gene expression changes, and suggest the novel important role of hypertrophic response modulation during different stages of cardiac remodeling.

## Introduction

The mechanism of transforming external stretch signals to a biochemical response in cytoplasmic elements and nucleus is one of the important components of cell adaptation and homeostasis control. This process, called mechanotransduction, plays an important role in different type of mechanosensing cells, and its dysregulation may consequently lead to progression of various diseases (Jaalouk and Lammerding, 2009). In cardiac and skeletal muscles mechanotransduction is used to adapt to external stimuli by changes in contractile and metabolic properties of muscle cells. In cardiomyocytes mechanosensing and mechanotransducing structures are concentrated in specific cell areas, and vast number of proteins and protein complexes involved in mechanical signal transmission are localized at sarcolemma, intercalated disks and sarcomere (Epstein and Davis, 2003; Lyon et al., 2015). The latter consists of myofilaments and large amount of interacting proteins that provide together the myofilament stability and integrity. One of the major part of sarcomeric architecture is the Z-disk – a relatively small macromolecular structure, containing the huge network of proteins which is proposed to serve as one of the key mechanosensing areas in cardiomyocytes. These proteins form the complex framework structure, which determinates contractile properties in normal conditions and under strain (Pyle and Solaro, 2004; Gautel and Djinovic-Carugo, 2016; Tabish et al., 2017). Except for the structural functions, Z-disk proteins are involved in transmembrane signaling, and activation of several stress-signal pathways in cytoplasm via interactions with kinases, phosphatases and through intracellular calcium signaling (Hoshijima, 2006; Luther, 2009). Mutations in genes encoding Z-disk components may lead to hereditary cardiac and muscle disorders such as cardiomyopathies and myopathies (Knöll et al., 2011; Kostareva et al., 2016; Schänzer et al., 2018). As a consequence, one of proposed molecular mechanisms for cardiomyopathies suggests that the impaired sarcomere stretch response is due to mutations in Z-band components (Kimura, 2016; Dal Ferro et al., 2017). Additionally, several newly described by genome-wide association studies loci related to dilated cardiomyopathy (DCM), eccentric heart remodeling and heart failure include Z-line genes (Villard et al., 2011; Garnier et al., 2015). However, exact mechanism of gene expression regulation under hemodynamic load and the detailed role of Z-line-associated proteins in this process are still not completely understood. In the present study, we used an *in vivo* model of myocardial hypertrophy caused by pressure overload to analyze time-dependent dynamics of Z-line-associated gene expression in different myocardial chambers (left ventricle, right ventricle, and interventricular septum). We demonstrated that several genes, encoding Z-disk proteins, have distinct mRNA expression patterns between free left ventricle wall and interventricular septum with the latter having expression profiles more similar to hemodynamically uncompromised right ventricle. The observed changes did not directly lead to modulation of protein expression, pointing on protein degradation pathways as an important regulatory mechanism in hemodynamic overload conditions.

## Materials and Methods

### Ethics Statement

All procedures were performed in accordance with the Guide for the Care and Use of Laboratory Animals published by the National Institute of Health and approved by the Local Ethics committees at Almazov National Medical Research Centre (Reference No. 16-3).

### Experimental Procedure of Pressure-Overload Hypertrophy

Eight-weeks-old male specific-pathogen-free Wistar rats (RRID:RGD_13508588) underwent banding of the aortic arch to perform pressure overload (Cho et al., 2014). During surgical procedures anesthesia was maintained with 1.8–2.2% isoflurane (“Foran,” Abbott Laboratories). Surgical access was performed via a left anterolateral thoracotomy in 4th intercostal space with partial resection of the 3rd rib in the area from parasternal to the midclavicular line. For the access to the aortic arch a small hole in the left lobe of the thymus formed by means of the blunt tissue separation was used. Banding was performed with 6-0 Prolene (Ethicon) ligation around the aortic arch and a 22 G blunted needle. In sham-operated groups similar surgical procedures were performed, only without aortic ligation. Due to severe pressure overload conditions post-operative mortality was 3.4%. Animals were divided into groups according to model duration – 1 week (*n* = 16), 2 weeks (*n* = 12), 8 weeks (*n* = 14), 10 weeks (*n* = 7). Intact (*n* = 9) and sham-operated animals [1 week (*n* = 8), 2 weeks (*n* = 3), and 8 weeks (*n* = 4)] were examined as control. During all period animals received food and water *ad libitum* and care was performed according to Good Laboratory Practice (GLP) standards.

After corresponding model duration animals were sacrificed under 2.2% isoflurane by removing hearts. Hearts was dissected from atria and vessels, heart apex was mounted into Frozen Section Medium (Richard-Allan Scientific Neg-5, Thermo Fisher Scientific), frozen by submerging in liquid nitrogen and stored at -80°C. Remaining heart tissue was divided into left and right ventricles, interventricular septum, and the parts were immediately frozen separately at -80°C until use.

### Echocardiography

During echocardiography rats were maintained with 1.5% isoflurane anesthesia. Ultrasound study was performed using Vevo-2100 (VisualSonics, Inc.) with scanning frequency 21 MHz and frame rate 100–120/s in 2D-mode. All measurements and calculations were performed according to recommendations from American Society of Echocardiography and the European Association of Cardiovascular Imaging (Lang et al., 2015). 2D- and M-images were obtained in longitudinal and short axes of the left ventricle (LV). M-mode cursor was positioned under control of 2D-imaging perpendicularly to left ventricle posterior wall (LVPW) and interventricular septum (IVS). Obtained images were analyzed offline. Left ventricle internal dimension (LVID), interventricular septal thickness at diastole (IVS_d_), left ventricle posterior wall thickness at diastole (LVPW_d_) were measured in two planes (short and long axes of left ventricle) in three cardiac cycles. Left ventricular mass (LVM) was calculated using mean value by Devereux and Reichek method (Devereux et al., 1986):

$$LVM(in\;mg)=0,8\times 1,04\times [{\left(IVS_{\text{d}}+LVPW_{\text{d}}+LVID_{\text{d}}\right)}^{3}-LVID_{\text{d}}^{3}]+0,6;$$

and left ventricle fractional shortening was calculated as

$$FS\%=[(LVID_{\text{d}}-LVID_{\text{d}})/LVID_{\text{d}}]\times 100,$$

where *d* and *s* indicate diastole and systole.

Left ventricle ejection fraction was calculated as

LVEF(%)=[EDV−ESV)/(EDV]×100,

where EDV is end-diastolic volume, ESV is end-systolic volume.

### Morphological Examination

12-μm thick cryostat sections of heart apices were prepared using cryotome The Tissue-Tek Cryo3 Flex (Sakura). Before staining sections were thawed and air dried for 30 min at room temperature. After blocking in 15% fetal calf serum (FCS) for 30 min at room temperature, samples were incubated overnight at +4°C with mouse monoclonal beta-sarcoglycan antibodies (Leica Biosystems, NCL-L-b-SARC). Signal was detected with DAB peroxidase substrate reaction using Novolink Polymer Detection System (Leica Biosystems) according to manufacturer’s recommendations. Sections were mounted with Richard-Allan Scientific Mounting Medium (Thermo Fisher Scientific). Imaging were acquired using light microscope Axio Observer D1 and ZEN software (Zeiss) with final magnification × 630. For analysis only cells with elliptical shape, distinct boundaries and approximately centrally located nucleus were chosen. Size of cardiomyocytes were quantified measuring minimal diameter of elliptical shaped cells in transverse section. Diameters were evaluated for 100 ± 20 cells from at least five microphotographs of an individual heart using NIH ImageJ software. Analysis was performed in a blind manner.

### RNA Isolation and Real-Time PCR

For total RNA isolation pieces of frozen samples were homogenized in TissueLyzer (QIAGEN) for 8 min at 50 Hz in Extract RNA reagent (Evrogen). Next purification steps were performed following instructions for Extract RNA reagent. Quality and purity of RNA were validated using NanoDrop 3300 SpectroPhotometer (Thermo Fisher Scientific) and electrophoresis in 1% agarose gel. RNA samples were treated with DNAseI (Thermo Fisher Scientific). cDNA template was synthesized using Random (dN)10-primer (Evrogen) and MMLV RT kit (Evrogen). Quantitative Real-Time PCR (qRT-PCR) was performed on cDNA template using commercial TaqMan Gene Expression Assays for *Nppa*, *Fhl1*, *Fhl2*, *Actn2*, *Synpo2*, *Myoz2*, *Nebl*, *Cmya5*, *Ldb3*, *Csrp3*, *Ilk*, and *Actb* genes (Applied Biosystems). Expression of *Hprt1* was evaluated by qRT-PCR using oligonucleotide gene-specific primer pair. The list of TaqMan assays and primers is provided in Table 1. mRNA relative expression was quantified applying _ΔΔ_C_t_-method using *Actb* and *Hprt1* as housekeeping control (Livak and Schmittgen, 2001).

**Table 1:** Oligonucleotide primers and Assay ID.

Target	Oligonucleotide primer/assay ID
*Hprt1* forw	5^′^-CAGGCCAGACTTTGTTGGAT-3^′^
*Hprt1* rev	5^′^-TCCACTTTCGCTGATGACAC-3^′^
*Actb*	Rn00667869_m1
*Nppa*	Rn00664637_g1
*Fhl1*	Rn01402101_m1
*Fhl2*	Rn00581565_m1
*Actn2*	Rn01470228_m1
*Nebl*	Rn01487582_m1
*Myoz2*	Rn01521544_m1
*Cmya5*	Rn01497633_m1
*Ldb3*	Rn01453313_m1
*Csrp3*	Rn00589257_m1
*Ilk*	Rn00591471_m1

### Immunoblotting

Frozen heart tissue samples were homogenized in TissueLyzer for 15 min at 50 Hz in lysis buffer [50 mM Tris-HCl pH 8.0, 1% Triton X-100, 0.1% sodium dodecyl sulfate (SDS), 150 mM sodium chloride] supplemented with protease inhibitors cocktail (Sigma-Aldrich), and soluble fraction of lysates was served. Protein concentration was measured using DC Protein Assay (Bio-Rad). Lysates were run in SDS- polyacrylamide gel. After transfer membranes were primary stained with 0.1% Ponceau S (Sigma-Aldrich) and further incubated with α-actinin (Santa-Cruz, Cat#sc-166524) or p62/SQSTM1 (Sigma-Aldrich, Cat# P0067) primary antibodies overnight. After incubation with spice-specific secondary antibodies (Immun-Star Goat Anti-Mouse (GAM) or Goat Anti-Rabbit (GAR) HRP Conjugate, Bio-Rad) and development with SuperSignal West Femto substrate (Thermo Fisher Scientific) chemiluminescence was detected using Fusion Fix (Vilbert Lourmat). To avoid the bias linked to uneven increase in protein content under myocyte hypertrophy, the normalization was performed using not a single house-keeping gene product such as GAPDH or β-actin, but a total protein level detected by Ponceau S staining and normalized as relative optical densities. Analysis was performed using ImageJ software.

### Statistical Analysis

Statistical analysis was performed in GraphPad Prism software. Normal distribution was tested using Shapiro–Wilk test. For paired comparison of normally distributed datasets Student’s *t*-test was used; for datasets that do not have normal distribution Mann–Whitney *U*-test was used. Relationship between LVM index and *Nppa* mRNA level in left ventricle was accessed by calculation of Pearson coefficient. Significant differences were considered if *P* < 0.05. All datasets are presented as dot-plot with mean ± standard deviation.

## Results

### Dynamics of Myocardial Hypertrophy in Aortic Constriction Model

**FIGURE 1:**
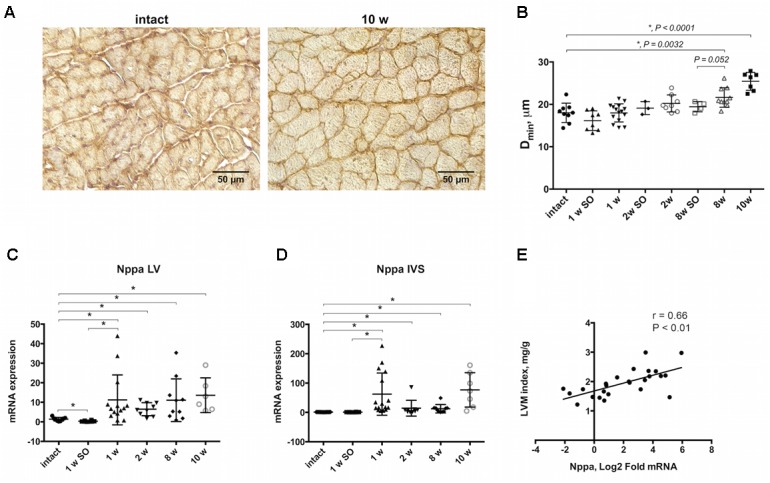
Morphological and molecular evidences of myocardial hypertrophy progression. **(A)** Immunohistochemical staining of beta- sarcoglycan allowing to detect outer membrane showing clear increase of cells size in transverse orientation by 10-weeks group compared to intact. **(B)** Increase in cell diameter (Dmin) illustrating progressive cardiomyocytes enlargement, and significant increase after 8 and 10 weeks of aortic banding performing. *Nppa* expression was upregulated in LV **(C)** and IVS **(D)** after 1, 2, 8, and 10 weeks of model duration compared to intact or sham-control groups. **(E)** Positive linear correlation was found for *Nppa* mRNA level and left ventricular mass (LVM) indexed to body weight. The analysis included experimental groups after 8 and 10 weeks of aortic constriction, 8 week’s sham-operated and intact animals, r indicates Pearson coefficient; for all ^∗^*P* < 0.05.

A rat model of cardiac hypertrophy was established by controlled constriction of the aortic arch. Myocardial weighing revealed significant increase in heart weight-to-body weight ratio by 10 weeks after experimental procedure compared to intact animals (3.48 ± 0.78 and 2.34 ± 0.20 correspondingly) (Table 2). Increase in LVM index along with no significant LVIDd increase confirmed the development of concentric hypertrophy by week 10 of aortic banding. Progression of cardiac remodeling was supported by strong tendency of LVPW increase after 8 and 10 weeks even without reaching a level of statistical significance. Similar tendency was also noted for IVS thickness. As expected, ejection fraction and fractional shortening decreased with time, again without reaching a statistical significance. Myocardial hypertrophy was additionally confirmed by histological analysis of heart tissue. Counting the minimal diameter (D_min_) of cardiomyocytes revealed continuous increase in myocyte size with significant increase after 8 and 10 weeks of the aortic banding (Figures 1A,B). To validate cardiac hypertrophy on molecular level we measured mRNA expression *Nppa*, encoding natriuretic peptides A, using qRT-PCR. Earlier, this gene was shown to be a reliable marker of cardiomyocyte hypertrophic response and activation of embryonic gene program (Sergeeva and Christoffels, 2013). Increased expression of *Nppa* was shown in LV and IVS myocardium on all time points of aortic constriction compared to intact and sham operated animals (Figures 1C,D). In right ventricle qRT-PCR analysis revealed low level of *Nppa* expression regardless of the time point and duration of aortic banding (data not shown). Furthermore, we observed a positive correlation between mRNA level of *Nppa* in LV and LVM index calculated by echocardiography (*r* = 0.66, *P* < 0.01) (Figure 1E). Thus, morphological examination and gene expression signature documented the development of myocardial hypertrophy by weeks 8–10 in current experimental model.

**Table 2:** Echocardiographic parameters obtained after different periods of aortic banding.

	Control			Time after aortic constriction			
	Intact (*n* = 5)	2 w SO (*n* = 3)	8 w SO (*n* = 4)	1 w (*n* = 10)	2 w (*n* = 8)	8 w (*n* = 10)	10 w (*n* = 6)
Body weight, g	326.0 ± 29.1	374.0 ± 29.4	380.8 ± 40.6	354.5 ± 33.3	337.6 ± 22.8	371.3 ± 29.0	347.0 ± 41.6
Heart wt-to-body wt ratio, mg/g	2.34 ± 0.20	2.47 ± 0.17	2.26 ± 0.34	2.53 ± 0.32	2.62 ± 0.46	2.76 ± 0.43	3.48 ± 0.78^∗^
LVM index, mg/g	1.70 ± 0.14	1.52 ± 0.22	1.44 ± 0.23	1.86 ± 0.17	1.94 ± 0.49	2.03 ± 0.43 ^†^	2.34 ± 0.40^∗^
FS, %	53.76 ± 0.82	54.22 ± 1.01	52.36 ± 4.13	46.29 ± 6.09	50.19 ± 7.82	47 ± 5.83	42.77 ± 10.92
LVEF, %	83.85 ± 0.80	85.47 ± 2.65	82.53 ± 3.02	76.14 ± 6.49	78.41 ± 7.2	76.28 ± 6.03	71.29 ± 12.41
IVS_d_, mm	1.40 ± 0.13	1.47 ± 0.09	1.42 ± 0.15	1.50 ± 0.11	1.47 ± 0.16	1.67 ± 0.25 (*P* = 0.073)	1.56 ± 0.20
LVPW_d_, mm	1.56 ± 0.11	1.52 ± 0.04	1.54 ± 0.17	1.64 ± 0.20	1.63 ± 0.21	1.84 ± 0.19 (*P* = 0.054)	1.90 ± 0.26 (*P* = 0.052)
LVID_d_, mm/g	1.91 ± 0.15	1.67 ± 0.15	1.63 ± 0.15	1.87 ± 0.22	1.94 ± 0.14^†^	1.76 ± 0.15	1.99 ± 0.30
RWT	0.48 ± 0.04	0.48 ± 0.05	0.48 ± 0.03	0.48 ± 0.07	0.47 ± 0.04	0.53 ± 0.08	0.51 ± 0.09

*SO, sham operated; wt, weight; LVM, left ventricular (LV) mass (indexed to body weight); FS, fractional shortening; LVEF, LV ejection fraction; IVS_d_, interventricular septum thickness; LVPW_d_, LV posterior wall; LVID_d_, LV internal dimension (indexed to body weight); RWT, relative wall thickness. Subscript d indicates diastole; P < 0.05 indicated vs. intact group (^∗^) and vs. corresponding SO group (^†^).*

### Expression Patterns of Genes, Coding Z-Disk Proteins, in Different Parts of Myocardium

**FIGURE 2:**
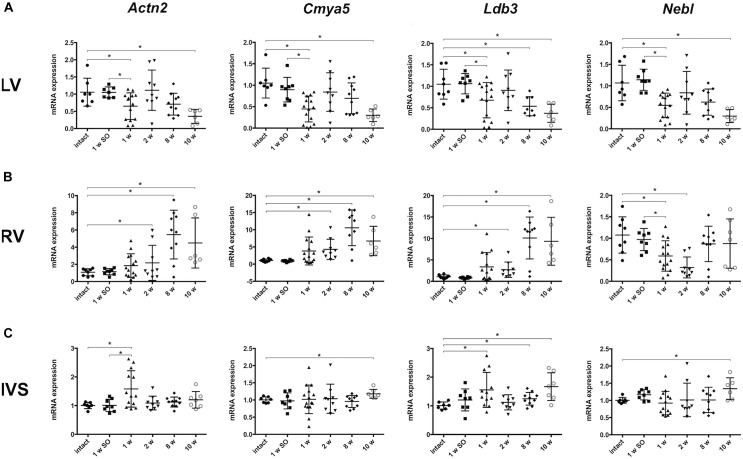
Altered mRNA expression of genes, encoding Z-disk proteins, in different parts of myocardium. **(A)** *Actn2*, *Cmya5*, and *Nebl* expression patterns after different period of aortic banding measured by qRT-PCR – downregulation after 1 week with subsequent return to normal level and further decrease after 10 weeks. Expression *Ldb3* declined after 1, 2, and 10 weeks of aortic banding. **(B)** Altered expression of genes, encoding Z-disk proteins, in right ventricle (RV). *Actn2*, *Cmya5*, and *Ldb3* expression was gradually increasing after 2 weeks up to 10 weeks of aortic constriction. Despite the gradual decrease of *Nebl* expression in RV, we showed no further *Nebl* reduction to the 10 week’s time point. Notably, expression pattern in RV is clearly distinct from pattern in LV. **(C)** Expression pattern of genes, encoding Z-disk proteins, in IVS are distinctly more close to RV than to LV profiles. *Cmya* and *Nebl* mRNA levels were elevated on later phases of hypertrophy progression (after 10 weeks). Opposite, *Actn2* is upregulated only on the early phase (after 1 week). *Ldb3* mRNA level was significantly increased after 1 week as well as after 10 weeks of aortic constriction. For all ^∗^*P* < 0.05.

Considering the role of Z-disk as mediator of external mechanical stimulus, we chose several genes, encoding both structural and signaling Z-disk-associated proteins (*Actn2*, *Ldb3*, *Cmya5*, *Fhl1*, *Fhl2*, *Csrp3*, *Nebl*, *Myoz2*), to assess gene expression profiles under pressure overload conditions at different time points of LV hypertrophy development. The Ilk-encoding gene was added this list as it was previously bioinformatically shown to be tightly linked to Z-disk-associated genes (Kostareva et al., 2016). Firstly, we studied time-dependent changes of mRNA level in left ventricular free wall myocardium (LV). Figure 2 illustrates that a set of genes – *Actn2* (encoding alpha-actinin-2), *Cmya5* (myospryn), *Ldb3* (LIM domain-binding protein 3) and *Nebl* (nebulette) – had similar expression profiles after aortic banding procedure. Particularly, expression of *Actn2*, *Cmya5*, and *Nebl* was significantly reduced after 1 week of aortic banding, normalized to control level by week 2 and subsequently decreased by weeks 8 and 10 (Figure 2A). Thus, we observed a significant decline in mRNA expression of some Z-disk associated genes at early stage after aortic banding and upon hypertrophy progression in LV free wall.

Analysis of pressure overload-induced gene expression changes in right ventricle revealed a pattern opposite to that observed in LV. A gradual increase of *Actn2*, *Cmya5*, and *Ldb3* expression was observed starting from week 1 to week 10 of experiment (Figure 2B). Of note, the early changes became significant 1 week later compared to LV (by experimental week 2), and expression of genes that were significantly downregulated in LV after 10 weeks, was strongly upregulated in RV at similar time point.

Further, we evaluated mRNA expression of analyzed genes, encoding Z-disk proteins, in IVS myocardial samples. We revealed that mRNA expression pattern of *Actn2*, *Cmya5*, *Nebl*, and *Ldb3* in IVS myocardium was more similar to the pattern in RV than LV free wall. In particular, *Cmya5*, *Ldb3*, and *Nebl* expression became elevated by 10 weeks of coarctation, while *Actn2* and *Ldb3* mRNA levels were significantly upregulated already after 1 week of aortic banding followed by reduction to normal level and increase at the later time points (Figure 2C).

Other genes selected for this study did not demonstrate defined change in expression pattern, and did not differ significantly between various time points and parts of myocardium (Supplementary Figure 1).

### Relation Between mRNA Level and Protein Expression in Different Parts of Myocardium Under Hemodynamic Pressure

**FIGURE 3:**
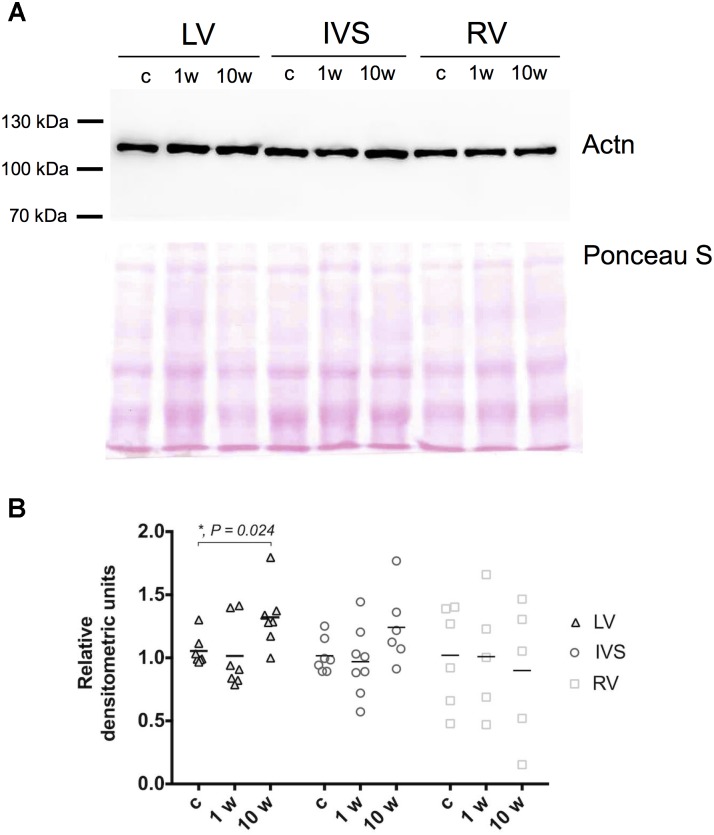
Expression of Actn in left, right ventricle and interventricular septum myocardium. Representative images of Western blotting of Actn2 in LV, IVS, and RV myocardium and total protein staining (Ponceau S) as loading control **(A)**. Densitometry analysis revealed significant increase of Actn2 expression only on 10 week’s time point in LV, and not in RV and IVS myocardium. Expression levels of Actn2 were normalized to Ponceau S staining and control group **(B)**. For all ^∗^*P* < 0.05.

To check whether observed alterations in gene mRNA expression lead to changes also on protein level we investigated the amount of Actn2 in myocardial tissue by immunoblotting at 1 and 10 week’s time points which correspond to maximum change on gene expression level. Contrary to reduction of *Actn2* mRNA in LV, Actn2 protein level demonstrated increase on 10 weeks after aortic banding compared to control animals (Figures 3A,B). In contrast, despite the increase of *Actn2* mRNA in IVS and RV, Actn protein expression did not significantly change at any investigated time points. Therefore, alteration of mRNA expression of Z-disk associated genes on early and later time points of myocardial hypertrophy does not always result in corresponding change in protein level, possibly due to modulation on protein turnover pathways. To check the possible involvement of protein turnover pathways in modulation of protein level under pressure overload conditions, p62 expression was evaluated in LV, RV and IVS myocardium by immunoblotting. We observed increase of p62 level after 10 weeks in LV myocardium, while no alterations were detected for RV and IVS (Supplementary Figure 2). Thus, changes in mRNA expression can be modulated through protein degradation system upon development of LV hypertrophy.

## Discussion

In cardiomyocytes mechanotransduction serves for transmission of the mechanical stimulus from membrane to the nucleus and involves several cell compartments – sarcolemmal protein complexes, intercalated disks, sarcomere and nuclear envelope (Lyon et al., 2015). Z-disks, located at the lateral borders of sarcomeres, are considered as one of the nodal points of signal transmission. A number of Z-associated proteins due to their domain organization may perform both structural and signaling properties and, thus, participate in regulation of gene expression (Frank et al., 2006). The potential signaling role of Z-disk is to perceive stress stimuli from sarcomere stretch and transmit it to the nucleus in order to adapt cardiomyocyte contractility, compliance and energetics to hemodynamic load (Hoshijima, 2006). Some of the proteins, initially located to Z-band region only, like muscle LIM protein (MLP) or myopodin (SYNPO2), can directly shuttle to the nucleus and enhance the action of transcription factors (Weins et al., 2001; Boateng et al., 2007).

In our study we identified time-dependent alterations of mRNA level of genes encoding Z-disk-associated proteins after induction of pressure-induced cardiac hypertrophy. We revealed that under LV pressure overload, expression of *Actn2*, *Cmya5*, *Ldb3*, and *Nebl* in LV myocardium was repressed at an early time point (1 week) with subsequent return to basal level, and thereafter again decrease at a late time point (10 weeks). Similar acute-phase regulation of cytoskeletal genes in LV was previously demonstrated by van den Bosch et al. (2006) in transverse aortic constriction (TAC) model. Together with our results, observed changes of transcript level of cytoskeletal and myofibrillar components in LV at the early time points may reflect an initial adaptive response for severe hemodynamic load.

We further detected the divergent gene expression profiles in left, right ventricles and interventricular septum during aortic constriction. As expected, after TAC gene expression patterns in RV and LV were different due to different hemodynamic stress. Surprisingly, IVS expression pattern of Z-disk associated genes was more similar to RV, rather than to LV. During embryonic heart development, LV and RV have a distinct cellular source: LV originates from primary heart field, whereas RV is developed from secondary heart field (Zaffran et al., 2004). Hemodynamic and molecular characteristics in RV stress response are less studied compared to LV (Voelkel et al., 2006). Transcriptome analysis in comparative investigations of pulmonary artery constriction (RV hypertrophy) and TAC (LV hypertrophy) revealed more abundant expression changes in RV than in LV and, moreover, underlined some ventricular-specific expression signatures of genes, responsible for cytoskeletal organization (Kreymborg et al., 2010; Friehs et al., 2013).

The development of IVS start from protrusions of both ventricles, and at a cellular level IVS has equal origin with contribution of cell growth from both LV and RV (Meilhac et al., 2004). Analysis of progenitor cardiac cells identified in the IVS demonstrated the presentation of this population as well in LV and notably not in RV, further supporting IVS similarity to LV origin (Stadtfeld et al., 2007). Increase of *Nppa* mRNA expression in IVS myocardium in our model confirms the activation of hypertrophic response program after TAC. However, expression pattern of genes, encoding for Z-disk proteins in IVS was more similar to RV rather than to LV. The latter fact suggests that the regulatory role of these genes differs in free LV wall and IVS. This observation might be important for unraveling molecular mechanisms of inherited cardiac hypertrophy where a single gene defect often leads to a marked difference in hypertrophic response in IVS and LV free wall. Thus, the obtained data may be of importance for understanding the molecular pathogenesis of hypertrophic cardiomyopathy (HCM) as well as other inherited cardiac pathologies. While the majority of HCM cases are caused by pathogenic variants in sarcomeric genes, the concomitant rare variants in structural and cytoskeletal genes and their expression profile may influence the phenotypic variability of cardiomyopathies (Lopes et al., 2015).

Changes of mRNA level can have impact not only on protein synthesis directly but also on regulatory processes. Previous investigation of transgenic mice with diastolic dysfunction revealed increase of desmin and α-actinin protein expression without altered transcriptional level (Sheng et al., 2016). In the present study, we also observed discrepancies in LV between mRNA and protein level of α-actinin at the late time point (10 weeks) of hemodynamic load. Observed divergence may partially be explained by concomitant increase of p62 protein level reflecting the repressed protein degradation (Klionsky et al., 2012) further supporting the possibility of regulatory rather than only structural role for Z-disk associated genes (Zhu et al., 2007; Gu et al., 2016). However, other protentional explanation may exist, for example, different life times of the molecules and different mechanisms of degradation.

In summary, we revealed that mRNA expression of genes encoding Z-disk proteins, is mostly reduced in LV myocardium on early and late phases of pressure overload hypertrophy. We demonstrated that expression levels of the same genes in RV have opposite to LV profile with IVS expression profile being more similar to RV rather than free LV. We observed that alteration in mRNA expression upon myocardial hypertrophy does not always correspond to protein level which could be modulated by protein turnover pathways and point on the fact that transcriptional changes do not entirely aim on adjusting protein level, but also on regulating the hypertrophic response during cardiac remodeling. Future integrated data using high throughput screening methods including RNA-sequencing and proteomic approach may provide further information about mechano-dependent gene expression profile in cardiovascular pathology.

## Author Contributions

AKn, AKr, AG, AM, GP, and AH performed the experiments. AKn, AKr, GP, and AH performed the statistical data analysis. AKo, AG, NS, and MG designed the study. AKn drafted the manuscript. TS, GS, and AKo revised and approved the final manuscript. All authors agreed to be accountable for all aspects of the work.

## Conflict of Interest Statement

The authors declare that the research was conducted in the absence of any commercial or financial relationships that could be construed as a potential conflict of interest.
