# Supplementary material for: Time- and Ventricular-Specific Expression Profiles of Genes Encoding Z-Disk Proteins in Pressure Overload Model of Left Ventricular Hypertrophy
Source: Front Genet. 2019 Jan 7;9:684. doi: 10.3389/fgene.2018.00684 (PMC6330284; doi:10.3389/fgene.2018.00684)
Supplement: Supplementary file 1 [file Presentation_1.PPTX]

## Slide 1
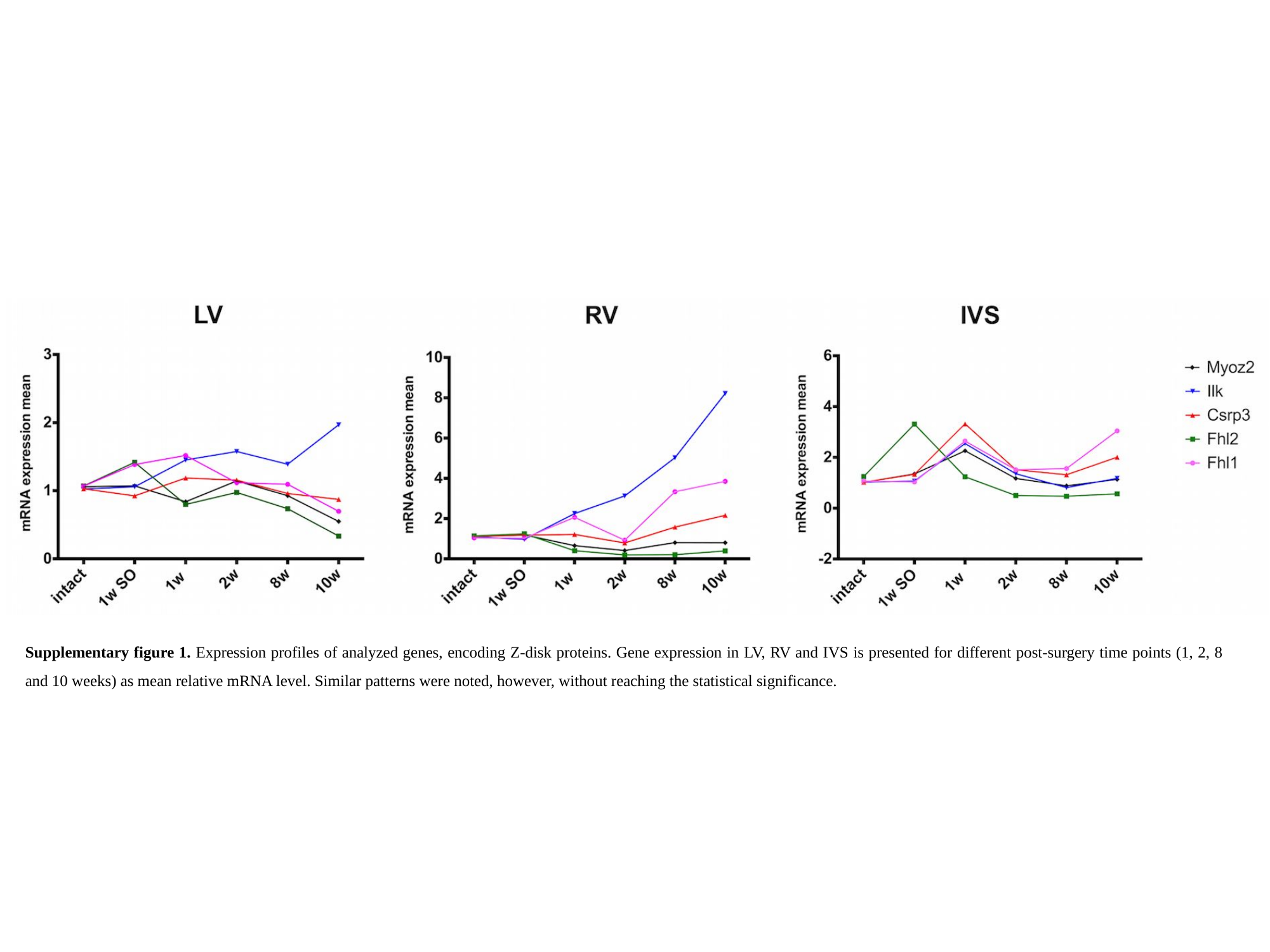

Supplementary figure 1. Expression profiles of analyzed genes, encoding Z-disk proteins. Gene expression in LV, RV and IVS is presented for different post-surgery time points (1, 2, 8 and 10 weeks) as mean relative mRNA level. Similar patterns were noted, however, without reaching the statistical significance.

## Slide 2
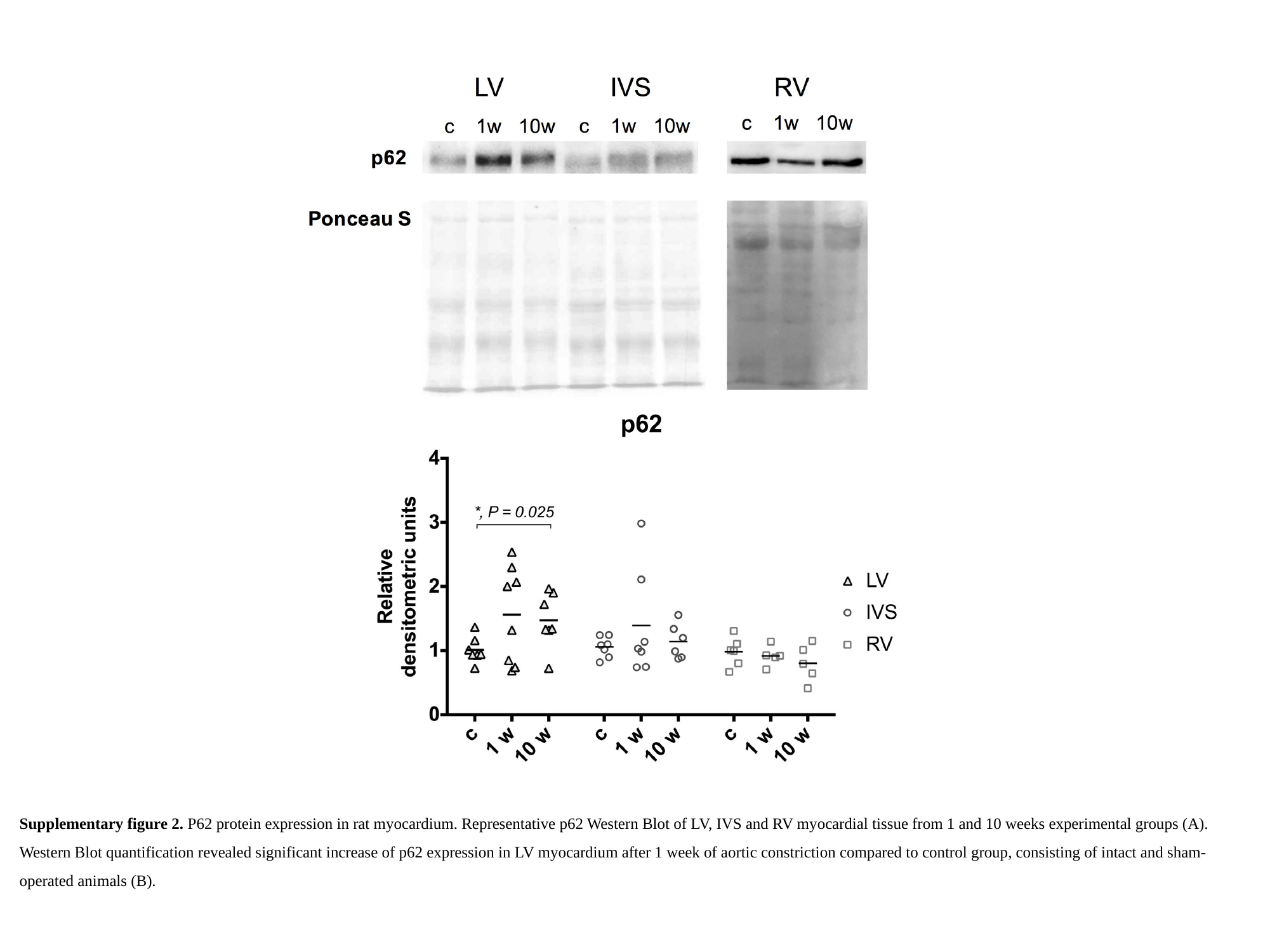

Supplementary figure 2. P62 protein expression in rat myocardium. Representative p62 Western Blot of LV, IVS and RV myocardial tissue from 1 and 10 weeks experimental groups (A). Western Blot quantification revealed significant increase of p62 expression in LV myocardium after 1 week of aortic constriction compared to control group, consisting of intact and sham-operated animals (B).
